# Essential thrombocythaemia progression to the fibrotic phase is associated with a decrease in JAK2 and PDL1 levels

**DOI:** 10.1007/s00277-022-05001-8

**Published:** 2022-10-21

**Authors:** Krzysztof Lewandowski, Zuzanna Kanduła, Michał Gniot, Edyta Paczkowska, Paulina Maria Nawrocka, Marzena Wojtaszewska, Michał Janowski, Magdalena Mariak, Luiza Handschuh, Piotr Kozlowski

**Affiliations:** 1 Department of Hematology and Bone Marrow Transplantation, Poznan University of Medical Sciences, Poznan, Poland; 2 Department of General Pathology, Pomeranian Medical University, Szczecin, Poland; 3 Laboratory of Genomics, Institute of Bioorganic Chemistry, Polish Academy of Sciences, Poznan, Poland; 4 Institute of Computing Science, Poznan University of Technology, 60-965 Poznan, Poland; 5 Institute of Bioorganic Chemistry, Polish Academy of Sciences, Poznan, Poland

**Keywords:** Essential thrombocythaemia, Post-essential thrombocythaemia myelofibrosis, *JAK2*V617F allele burden, *JAK2*V617F expression, Total *JAK2* expression, PDL1 expression, Driver defects, Non-driver defects, Inflammation

## Abstract

It has been postulated that the changes in the molecular characteristics of the malignant clone(s) and the abnormal activation of JAK-STAT signaling are responsible for myeloproliferative neoplasm progression to more advanced disease phases and the immune escape of the malignant clone. The continuous JAK-STAT pathway activation leads to enhanced activity of the promoter of CD274 coding programmed death-1 receptor ligand (PD-L1), increased PD-L1 level, and the immune escape of MPN cells. The aim of study was to evaluate the *PDL1* mRNA and *JAK2* mRNA level in molecularly defined essential thrombocythaemia (ET) patients (pts) during disease progression to post-ET- myelofibrosis (post-ET-MF). The study group consisted of 162 ET pts, including 30 pts diagnosed with post-ET-MF. The *JAK2*V617F, *CALR*, and *MPL* mutations were found in 59.3%, 19.1%, and 1.2% of pts, respectively. No copy-number alternations of the *JAK2*, *PDL1*, and *PDCDL1G2* (*PDL2*) genes were found. The level of PD-L1 was significantly higher in the *JAK2*V617F than in the *JAK2*WT, *CALR* mutation-positive, and triple-negative pts. The PD-L1 mRNA level was weakly correlated with both the *JAK2*V617F variant allele frequency (VAF), and with the JAK2V617F allele mRNA level. The total *JAK2* level in post-ET-MF pts was lower than in ET pts, despite the lack of differences in the *JAK2*V617F VAF. In addition, the PD-L1 level was lower in post-ET-MF. A detailed analysis has shown that the decrease in *JAK2* and *PDL1* mRNA levels depended on the bone marrow fibrosis grade. The *PDL1* expression showed no differences in relation to the genotype of the *JAK2* haplotype^GGCC_46/1^, hemoglobin concentration, hematocrit value, leukocyte, and platelet counts. The observed drop of the total *JAK2* and *PDL1* levels during the ET progression to the post-ET-MF may reflect the changes in the *JAK2*V617F positive clone proliferative potential and the PD-L1 level–related immunosuppressive effect. The above-mentioned hypothesis is supported by The Cancer Genome Atlas (TCGA) data, confirming a strong positive association between *CD274* (encoding PD-L1), *CXCR3* (encoding CXCR3), and *CSF1* (encoding M-CSF) expression levels, and recently published results documenting a drop in the CXCR3 level and circulating M-CSF in patients with post-ET-MF.

**Supplementary Information:**

The online version contains supplementary material available at 10.1007/s00277-022-05001-8.

## Introduction

Activation of the Janus kinase, a signal transducer and activator of transcription (JAK-STAT) signaling pathway in myeloid progenitor cells, is a hallmark of Philadelphia negative myeloid neoplasms (MPNPh −). It resulted in the proliferative advantage of myeloid cells, leading to clinical and laboratory symptoms of MPN [1, 2]. In the majority of MPNPh- patients (pts), clonal proliferation of myeloid progenitor cells is the result of the mutation acquisition by hematopoietic stem cells in the gene coding proteins of the JAK-STAT signaling pathway [3]. In 2005, a gain-of-function point mutation in Janus 2 kinase gene (*JAK2*) was discovered and characterized by a few independent scientific groups [4–6]. The presence of another somatic activating mutation of the thrombopoietin receptor gene (*MPL* proto-oncogene) was confirmed by Pikman et al. and, independently, by Pardanani et al. in 2006 [7, 8]. The most frequent mutations affect the hotspot codon W515 (W515L/R/A/G) which is localized at the boundary of the transmembrane and the cytosolic domains of MPL [9]. W515 prevents spontaneous activation of MPL, but in the case of gain-off-function *MPL* mutations, the TPO-independent activation of the receptor takes place, resulting in the downstream JAK-STAT pathway signaling [10]. In 2013, the whole-exome sequencing studies confirmed the presence of recurrent frameshift mutation in the calreticulin gene (*CALR*) in ET pts negative for *JAK2* and *MPL* mutation [11, 12]. Until now, more than 60 mutations in *CALR* in MPN pts have been identified. Eighty percent of them are type 1 (a 52-bp deletion; c.1099_1150del, p.Leu367Thrfs*46) or type 2 mutations (a 5-bp insertion; c.1154_1155insTTGTC, p.Lys385Asnfs*47). The rest of the *CALR* gene mutations can be categorized into type-1-like, type-2-like, or other types [13]. According to recent data, the distribution of *CALR* mutations varies dependently from the type of MPNPh − . In ET, most patients harbor a mutation in one of three genes: JAK2 V617F (in 60%), CALR (in 20%), or MPL in (3%), with the distribution of type 1 and type 2 mutations being similar (51 vs. 39% of pts, respectively) [14–16]. The CALR mutants form a molecular complex with thrombopoietin receptor, determining a cytokine-independent JAK-STAT activation with an increased megakaryocyte proliferation [17, 18]. Recently, it has been postulated that defective interactions of mutant CALR with store-operated calcium (Ca2 +) entry machinery (SOCE) are responsible for (i) constitutive activation of SOCE, (ii) MPL activation, and (iii) subsequent phosphorylation of STAT5, AKT, and ERK1/2 and (iv) megakaryocyte proliferation [15]. In the case of the MPL gene mutations, coding the thrombopoietin receptor structure, the constitutive activation of thrombopoietin receptor activation is responsible for JAK-STAT pathway activation [9].

The proliferation and differentiation of MPNPh − cells due to abnormal JAK-STAT pathway signaling results in an indirect paracrine secretion of inflammatory cytokines released by the bone marrow microenvironment cells and the cytokine storm. One of the consequences is bone marrow fibrosis associated with an increased level of interleukin 8 (IL-8), oncostatin-M, lipocalin-2, transforming growth factor β1, platelet derived growth factor (PDGF), fibroblast growth factor (FGF), venous endothelial growth factor (VEGF), and inhibitors of matrix metalloproteinases in the peripheral blood [19–21]. The frequency of fibrotic transformation of ET to post-essential thrombocythaemia myelofibrosis (post-ET-MF) was determined as 0.8–4.9% at 10 and 4–11% at 15 years, respectively [22]. According to the available data, the cumulative risk of post-ET-MF increases over time and is 0.3% at 5 years and 3.9% at 10 years, with the median follow-up time of 9.1 years [23]. Pre-fibrotic primary myelofibrosis bone marrow morphology, advanced age, and anemia were identified as factors predisposing to post-ET-MF. The presence of the *JAK2* mutation was associated with a low fibrotic transformation risk of ET [24–26]. It has been also postulated that *JAK2*V617F variant allele frequency (VAF) is correlated with fibrotic progression [27].

Hao et al. and, independently, Barrett et al. postulated a possible relation between the expression of *JAK2*, and the PD-1 ligand genes [*PD-L1* (*CD274*) and *PD-L2* (*CD273*)], because all of them are located on chromosome 9p24.1, and an amplification of chromosome 9p24.1 upregulated the *JAK2* expression and activated the JAK2-STAT3 pathway [28, 29]. It was found that *JAK2*V617F oncogenic activity resulted in an increased phosphorylation of STAT3 and STAT5 and enhanced PD-L1 promotor activity and PD-L1 protein level [30]. The upregulation of PD-L1 resulted in a reduced cytotoxic T cell activity, cell cycle progression, and T cell exhaustion [31, 32]. Recently, Milosevic-Feenstra et al. documented a higher PD-L1 mRNA expression in granulocytes in both *JAK2*V617F positive ET and primary myelofibrosis (PMF) patients, compared to *CALR*-mutated MPN patients. Moreover, they showed that MPN cells in *JAK2*-V617F-positive patients expressed higher levels of PD-L1, if 9p uniparental disomy (UPD) was present [33]. The aforementioned mechanism may confer a potent escape mechanism of MPN cells from the host immune system and disease progression. Therefore, our study aimed to evaluate the PD-L1 and JAK2 mRNA expression in molecularly defined ET patients, dependently from the disease phase, to answer the question of the mutual relation between the PD-L1mRNA and JAK2 mRNA expression and disease progression to post-ET- MF.

## Materials and methods

### Study group characteristics

The study group consisted of 132 pts with ET and 30 persons with a confirmed diagnosis of post-ET-MF according to the WHO criteria published in 2016 [34]. All patients were reevaluated for the presence of symptoms suggesting pre-fibrotic myelofibrosis or primary myelofibrosis, according to the criteria proposed by Barosi G et al. at the final step of the qualification process for the study [35]. The pts were recruited from two academic centers — the Department of Hematology and Bone Marrow Transplantation of Poznan University of Medical Sciences in Poznan, Poland, and the Department of Hematology and Department of General Pathology of Pomeranian Medical University in Szczecin, Poland. The duration of the follow-up and the type of cytoreductive treatment used in patients with ET and subjects with ET transforming to the post-ET-MF is presented in Table 1. The clinical patient workup included physical examination, ultrasonography, magnetic resonance, and computed tomography imaging. The diagnostic algorithm also took into consideration peripheral blood and bone marrow biopsies analysis, trephine bone marrow biopsy assessment, and molecular testing for the *BCR-ABL* fusion gene, as well as *JAK2*, *CALR*, and *MPL* mutation screening. All patients studied have been monitored regularly (every 1–3 months) in Outpatient Department. In the case of symptoms suggesting ET transformation to post ET-MF, a trephine biopsy was performed to confirm the fibrotic transformation. In all these patients, repeated analysis of the initial trephine biopsy specimen was performed (if available).

**Table1:** The duration of the follow-up and the type of cytoreductive treatment used in patients with ET and subjects with ET transforming to the post-ET-MF

Age (y)/number	Study group (*n* = 162, male/females = 61/101 (38%/62%), median age (y, range = 62 (22–95))													
	ET (*n* = 132)							Post-ET-MF (*n* = 30)						
	Follow-up duration (y, median)	No Tx	HC	INF	HC/INF*	HC/ANA*	HC/ANA/INF*	Follow-up duration (y, median)	No Tx	HC	INF	HC/INF*	HC/ANA*	HC/ANA/INF*
< 40 (*n* = 29)	6.8	17	2	1	0	2	1	9.2	2	0	0	2	2	0
40–60 (*n* = 48)	5.5	21	17	0	0	3	0	10.3	2	4	0	0	1	0
> 60 (*n* = 85)	4.6	14	45	1	1	6	1	4.8	7	9	0	1	0	0

*n* number of patients, *ET* essential thrombocythaemia, *No Tx* no therapy, *HC* hydroxycarbamide, *ANA* anagrelide, *INF* PEG-interferon 2α

^*^Cases treated with sequential therapy due to treatment failure or intolerance

The criteria for the diagnosis of post-ET-MF was based on the consensus statement from the International Working Group for Myelofibrosis Research and Treatment and included the documentation of a previous diagnosis of ET, as defined by the WHO criteria, as well as the confirmation of bone marrow fibrosis grades 2–3 (on 0–3 scale), and at least two of the following criteria: anemia and a ≥ 2-g/dL decrease from baseline hemoglobin level, a leucoerythroblastic peripheral blood picture, increasing splenomegaly, defined as either an increase in palpable splenomegaly of ≥ 5 cm (the distance of the tip of the spleen from the left costal margin), or the appearance of newly palpable splenomegaly, increased lactic dehydrogenase activity above the reference level, the development of ≥ 1 of three constitutional symptoms: > 10% weight loss in 6 months, night sweats or unexplained fever (> 37.5 °C) [36, 37]. The grade of the bone marrow fibrosis was assessed according to the European consensus on grading bone marrow fibrosis and the assessment of cellularity [38].

### Methods

DNA and RNA were extracted from whole-blood leukocytes at the time of the initial evaluation due to the clinical suspicion of ET or disease evolution to the fibrotic phase. Genomic DNA was isolated using the QIAamp® DNA Blood Mini Kit (QUIAGEN). Total RNA was extracted with TRIzol™ (Invitrogen). Purity and quantity of DNA and RNA were assessed with NanoDrop 1000 Spectrophotometer (Thermo Fisher Scientific).

The assessment for the presence of the *JAK2*V617F mutation was conducted by a quantitative allele-specific PCR (ASO-PCR) according to Larsen et al. [39], and standardized in cooperation with MPN&MPNr EuroNet [40]. A high-resolution melting analysis (HRMA) was used to detect mutations in *CALR* (exon 9), *MPL* (exon 10), *SRSF2* (exon 1), and *U2AF1* (exons 2 and 6) genes, as previously described by Klampfl et al. [12], Boyd et al. [41], Lin et al. [42], and Qian et al. [43], respectively. For the identification of the mutation type identified by HRMA, the Sanger sequencing was applied using the BigDye Terminator v3.1 Cycle Sequencing kit (Applied Biosystems, Thermo Fisher Scientific). The sequence of exon 13 (range Ile574 to Glu727) of the *ASXL1* gene (a region covering at least 83% of all known *ASXL1* mutations), was analyzed by Sanger sequencing [44, 45]. To determine the *JAK2* haplotype^GGCC_46/1^, the rs12343867 SNP was genotyped [46].

The analysis of the *JAK2*WT and *JAK2*V617F mRNA level and PD-L1 mRNA level was done with the help of methods presented in the Supplementary File Methods. The gene copy number analysis (CNA) was performed with the use of the in-house developed multiplex ligation-dependent probe amplification (MLPA) test (Supplementary File Methods), designed and executed according to the well-established protocol, described before [47, 48].

### Statistical analysis

All basic statistical analyses were performed in Statistica 13 [TIBCO Software Inc. (2017). Statistica (data analysis software system), version 13, www.statsoft.pl/statistica-i-tibco-software/. Depending on the distribution of the variables, a proper parametric (ANOVA F) or non-parametric (Mann–Whitney, Kruskal–Wallis) test was used. In the case of variance and correlation study, an analysis was performed in R ver. 4.0.4 and R Studio ver. 1.4.1106, with the following R packages: base, ggcorrplot, dplyr, and Hmisc. For the association of gene expression with the mutation status and clinical features, ANOVA and MANOVA tests were applied, for univariate and multivariate analysis of variance, respectively. Because the data did not follow a normal distribution, Spearman’s rank correlation coefficients were calculated to estimate the correlation between the studied gene expression levels and blood parameters. Information on protein–protein interactions and the co-expression score, based on the RNA expression pattern, was obtained from the STRING database v. 11.5 (https://string-db.org).

## Results

### Study group characteristics

A detailed characteristic of the studied ET pts (*n* = 162) is presented in Table 1. According to our data, the frequency of the fibrotic transformation in patients over 60 years old was 20% (17/85), in the group of individuals between 40 and 60 years old 14.6% (7/48), and in patients < 40 years old 20.7% (6/29). In reported individuals experiencing ET progression to the fibrotic phase, the median follow-up duration was as long as ~ 10 years, with the exception of patients > 60 years old (~ 5 years). For ET diagnosis confirmation, trephine biopsy was performed at the time of diagnosis in 132 out of 162 cases (in the other cases, only aspiration biopsy was performed). Repeated biopsy was performed in 32 patients with clinical and/or laboratory symptoms suggesting a possible disease transformation to post-ET-MF. In thirty of them, post-ET-MF was diagnosed. In our resources, there was no data concerning the reclassification of patients diagnosed with ET to pre-fibrotic primary myelofibrosis or primary myelofibrosis.

The comparison of the different molecularly defined subgroups showed that the hemoglobin blood concentration was significantly increased in the *JAK2*V617F-positive pts (ANOVA test, *p* = 0.0165; Supplementary File Results). On the contrary, there was no difference in the hemoglobin blood concentration between ET and post-ET-MF pts (Kruskal–Wallis test, *p* = 0.0843; Supplementary file Results). An analysis of the WBC count in relation to the *JAK2*V617F VAF (< 50% and ≥ 50%) confirmed increased WBC count in pts with VAF ≥ 50% (Kruskal–Wallis test, *p* = 0.0018; Supplementary file Results). The comparison of the platelet count in molecularly defined ET subgroups showed that the platelet count was significantly higher only in the *CALR*-mutation-positive vs. *CALR*-mutation-negative pts (Mann–Whitney test, *p* = 0.0219; Supplementary file Results). *JAK2*V617F mutation was present in 96 (59.3%), *CALR* mutation in 31 (types 1–12, types 2–15, others 4, overall 19.1%), and *MPL* mutations in two (1.2%) out of the 162 pts. The co-occurrence of *JAK2*V617F and either *CALR* or *MPL* mutation was identified in three (1.9%) and 1 (0.6%) of the pts, respectively. Twenty-nine out of the 162 (17.9%) analyzed pts were triple-negative. The *ASXL1*, *SRSF2*, and *U2AF1* mutations co-occurred in 7/96 (7.3%) pts with *JAK2*V617F and 3/31 (9.7%) pts with the *CALR* mutations. Thirteen non-driver mutations (*ASXL1*, *SRSF2*, *U2AF1*) were present in 11/162 (6.8%) of the pts. The distribution of the non-driver gene mutations and molecular characteristics of ET and post-ET-MF pts harboring two or more mutations are shown in Supplementary file Results.

### ET and JAK2 haplotype GGCC_46/1, driving mutations, and JAK2 variant allele frequency

The study of the *JAK2* haplotype^GGCC_46/1^ (tagged by the C allele of rs12343867 SNP) showed the C/C genotype in 31 (19%), C/T in 73 (45%), and T/T in 58 (36%) pts. An analysis of the frequency of different *JAK2 haplotype*^*GGCC_46/1*^ genotypes in *JAK2*V617F positive ET pts showed a higher frequency of the mutation in the C/C vs. C/T and T/T haplotype carriers (Fisher exact test, *p* = 0.0228). No association of the *JAK2* haplotype^GGCC_46/1^ was observed with the *CALR* mutations (data not shown). The *JAK2*V617F VAF distribution between different *JAK2 haplotype*^*GGCC_46/*1^ groups showed that the allelic burden was significantly higher in the C/C than in other genotype carriers (*p* = 0.0198; Supplementary file Results). Moreover, a significant increase of C/C genotype percentage was observed in the ET pts with *JAK2*V617F VAF > 50% (*p* = 0.0033; Supplementary file Results). No distribution differences were noticed in terms of the WBC, platelet count, hemoglobin concentration, and age at diagnosis in different *JAK2* haplotype^GGCC_46/1^ groups.

### Gene copy number aberrations

No copy-number alternations of the *JAK2*, and *CD274* and *PDCDL1G2* gene (encoding proteins PD-L1 and PD-L2, respectively) located in close proximity to *JAK2* at the 9p24 chromosome locus was detected with the use of the in-house designed MLPA assay (Supplementary File Methods).

### JAK2V617F variant allele frequency and expression

*JAK2*V617F VAF was determined by the ASO-PCR and MLPA technique (*r* = 0.9217). Low and high *JAK2*V617F allele burden was found in 81/96 (84.4%) and 15/96 (10.4%) of the pts, respectively. In the *JAK2*V617F positive ET pts, the *JAK2*V617F allelic mRNA level was positively correlated with the *JAK2*V617F VAF (*r* = 0.6337; Supplementary file Results). The analysis of the *JAK2*V617F mRNA showed the presence of a low-level *JAK2*V617F allele in four additional individuals in which the mutation was not detected by a DNA analysis with ASO-PCR and MLPA. The total *JAK2* level in *JAK2*V617F positive pts was higher than in *JAK2*WT individuals (*p* = 0.0130; Supplementary file Results).

*JAK2*V617F VAF was higher in pts with leukocytosis at the time of evaluation (*p* = 0.0015). In addition, the WBC count was higher in ET pts with high *JAK2*V617F VAF (Supplementary file Results). An analysis performed in pts with low (≤ 50%) and high (> 50%) *JAK2*V617F VAF did not show any differences in the mean values of hemoglobin concentration and platelet count in the blood. Pts carrying the *CALR* mutation had significantly higher median platelet count in comparison to the *CALR* mutation-negative pts (ANOVA test, *p* = 0.028; Supplementary file Results). A similar analysis was not performed for the *MPL* mutated pts due to an insufficient number of positive pts (*n* = 3).

The total *JAK2* level did not differ between the different genotypes of the *JAK2* haplotype^GGCC_46/1^. No male/female differences in the *JAK2* level (both total, in all pts, and *JAK2*V617F positive pts) were observed.

### PD-L1 and JAK2 expression in the molecularly defined essential thrombocythaemia dependently from the bone marrow fibrosis grade

**Fig. 1:**
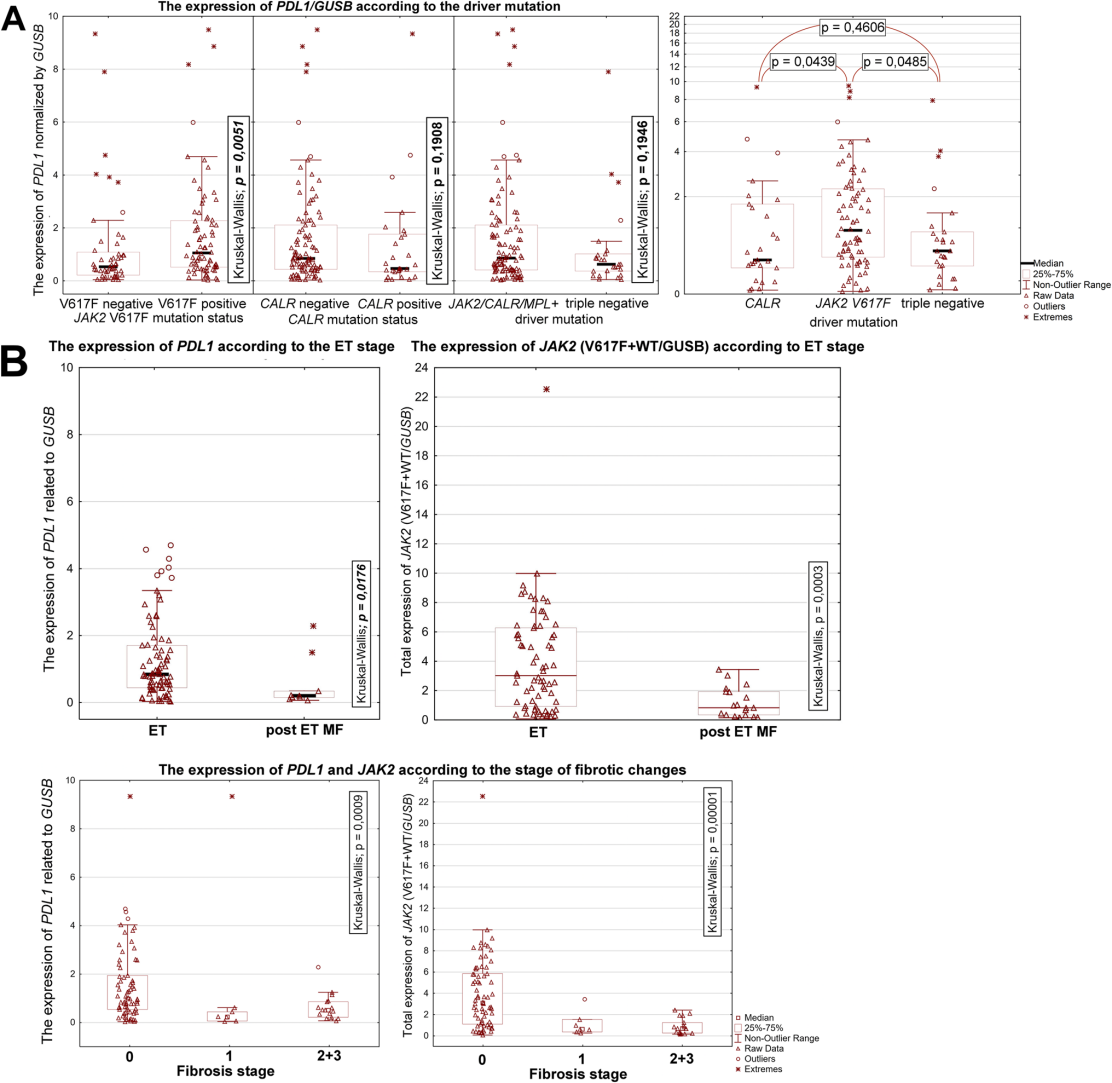
The mRNA expression of programmed death ligand 1 (PD-L1) and Janus tyrosine kinase 2 (JAK2 (WT + V617F)), dependently from the ET driver mutation status (**A**) and the grade of myelofibrosis (**B**)

The total *JAK2* expression level (*JAK2*V617F + wild type) showed no differences depending on the types of driving mutation (Kruskal–Wallis test, *p* = 0.1085; Supplementary file Results). The expression of PD-L1 was significantly higher in the *JAK2*V617F mutation-positive vs. *JAK2*WT ET pts (Kruskal-Willis test, *p* = 0.0051) (Fig. 1). A similar analysis performed in the *CALR* mutation-positive and *CALR*WT pts showed no differences (Kruskal-Willis test, *p* = 0.1908; Fig. 1). There were no differences in the expression of PD-L1 in the *JAK2/CALR/MPL* mutation-positive and triple-negative ET pts. The PD-L1 expression in the *JAK2*V617F positive pts was higher than in the *CALR* mutation-positive and triple-negative pts (Kruskal-Willis test, *p* = 0.0439 and *p* = 0.0485, respectively; Fig. 1).

An analysis performed independently from the driver mutation status showed that the PD-L1 mRNA level was significantly lower in the post-ET-MF than ET pts (Kruskal-Willis test, *p* = 0.0176; Fig. 1).

**Fig. 2:**
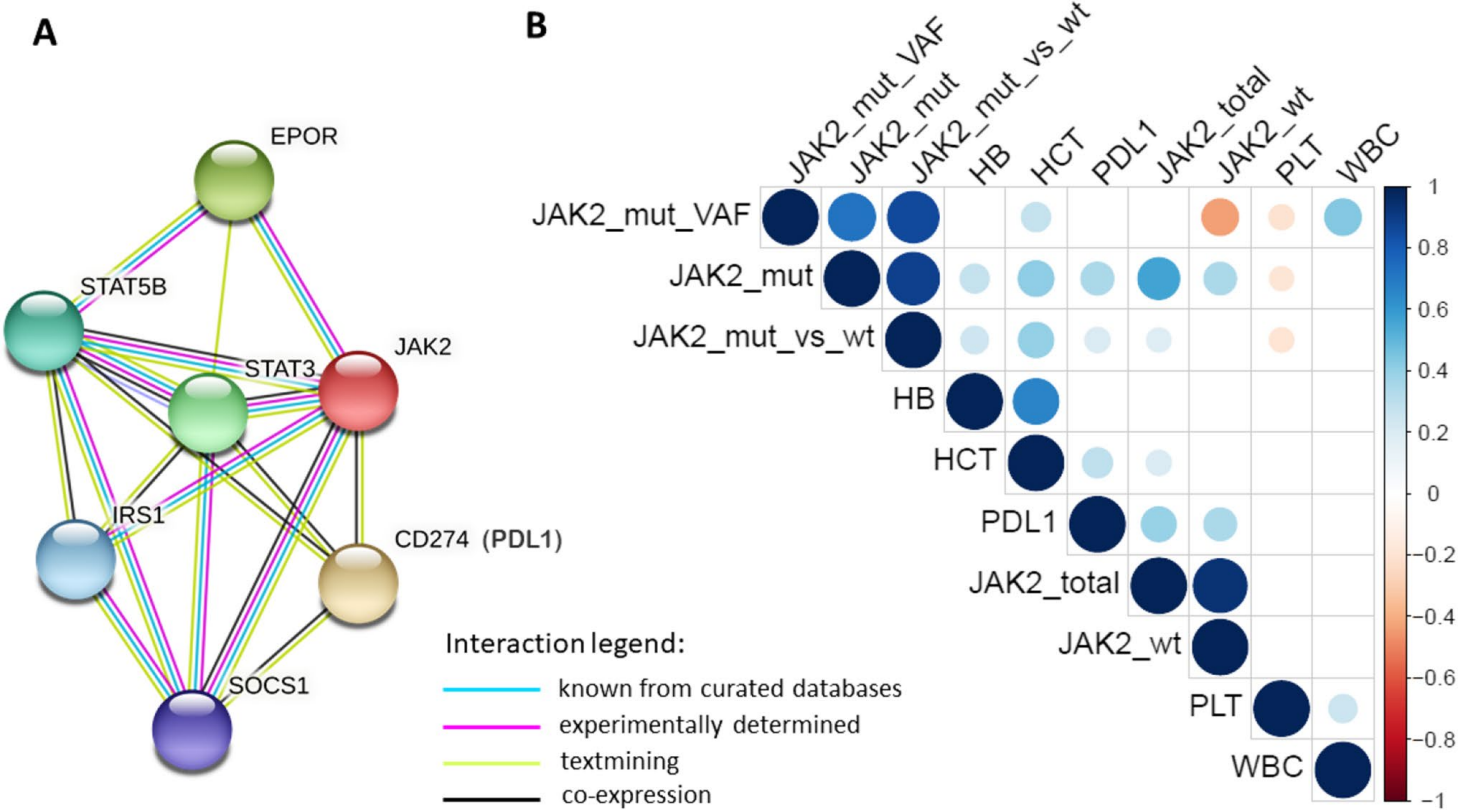
The association between the expression of *PDL1* and *JAK2* (both, V617F mutated and wild type allele (wt)) and complete blood count results in the studied cohort of ET patients. **A** The analysis of relations between genes encoding JAK2 and CD274 (PDL1) proteins. The network of five proteins interacting with JAK2 and CD274 (PDL1) generated by STRING v. 11.5 (https://string-db.org). RNA co-expression score observed in *Homo sapiens* for JAK2 and CD274 was 0.097. **B** A graphical presentation of Spearman correlation of *JAK2* and *PDL1* gene expression levels and blood parameters measured for patients included into the study. The color intensities reflect the correlation coefficients according to the legend on the right side of the graph (blue circles mean positive correlations, and red circles mean negative ones). The size of the circles is proportional to the level of significance (only statistically significant correlations are presented, with the *p* value threshold set as 0.05). Abbreviations: JAK2_mut_VAF, *JAK2* V617F variant allele frequency; JAK2_mut, expression of mutated *JAK2* allele; JAK2_wt, expression of wild type *JAK2* allele; JAK2_mut_vs_wt, the ratio of expression of mutated *JAK2* allele versus wild-type *JAK2* allele; JAK2_total, total *JAK2* expression (the sum of both alleles); PDL1, expression of *PDL1* (*CD274*) gene; HB, hemoglobin; HCT, hematocrit; PLT, platelets; WBC, white blood count

In addition, the total JAK2 level in post-ET-MF pts was lower than in ET pts without fibrotic transformation (Kruskal Willis test, *p* = 0.0003). It was evident, despite the lack of differences between the *JAK2*V617F VAF in ET and post-ET-MF pts (Kruskal–Wallis test, *p* = 0.3785; Supplementary file Results). A detailed analysis has shown that the decrease in JAK2 and PD-L1 mRNA level was gradual, depending on the bone marrow fibrosis grade (MF grade 0 vs 1 vs 2 *p* < 0.001, respectively; Fig. 1). A weak correlation was observed in the case of the PD-L1 and JAK2 total (JAK2V617F + JAK2WT) mRNA level (*r* = 0.1259, data not shown). Moreover, a low correlation between the PD-L1 mRNA level and *JAK2*V617F mutation VAF or the *JAK2*V617F allele mRNA level was found (*r* = 0.204; *p* = 0.049 and *r* = 0.232; *p* = 0.024, respectively). The correlation between the expression of PDL1 and JAK2 (both, mutated and WT allele) (Fig. 2B) observed here corresponds with the data retrieved from the STRING database v. 11.5 (https://string-db.org) [49]. According to STRING, the predicted JAK2 and PDL1 protein interaction is supported by the co-expression of the genes encoding both proteins (Fig. 2A; RNA co-expression score 0.097).

The study of the relationship between the *PD-L1* expression and *JAK2* haplotype^GGCC_46/1^ genotype, hemoglobin concentration, hematocrit value, leukocyte, and platelet count showed no differences (data not shown).

## Discussion

In 2008, Pardanani et al. based on an analysis of SNPs in four candidate genes (*EPOR*, *MPL*, *GCSFR*, *JAK2*) confirmed a significant association between the specific SNPs in the *JAK2* gene (46/1 haplotype or GGCC haplotype) and the onset of sporadic MPNs [50]. The allele frequency of the *JAK2* haplotype^GGCC_46/1^ in the healthy population is about 24%. Its presence is significantly increased (40 to 80%) in *JAK2*V617F positive MPN pts [51–55].

In our study, we showed that the *JAK2* haplotype^GGCC_46/1^ was significantly more frequent in *JAK2*V617F-positive than in *CALR*-positive ET pts. The latter observation is in agreement with the data previously reported by others [56].

Our results confirmed a significantly higher *JAK2*V617F VAF in homozygous carriers of *JAK2* haplotype^GGCC_46/1^ (C/C genotype) and a significant increase of the C/C genotype in ET pts with *JAK2*V617F VAF > 50%.

The total *JAK2* mRNA level did not significantly differ between pts defined by *JAK2* haplotype^GGCC_46/1^, but it was significantly increased in *JAK2*V617F positive pts. In our opinion, the increased total *JAK2* expression in ET pts may result from different factors, including the allelic expression imbalance of *JAK2* V617F mutation, MPN-associated chronic inflammation, the presence of other non-coding SNP affecting the *JAK2* expression, or mutations of epigenetic genes regulators (DNMT3A, TET2, EZH2, ASXL1, and IDH1/2 (via effects on TET2-mediated methylation)).

In 2013, Kim et al. demonstrated the *JAK2*V617F allelic imbalance leads to an increase of mutant allele, especially in ET (threefold increase) and polycythaemia vera (PV) (twofold increase) pts. Another possibility of an increased total *JAK2* mRNA expression in ET pts is abnormal JAK-STAT signaling. The potential link between a chronic inflammation and the development of myeloproliferative neoplasm has been postulated by Hasselbalch in 2012 [57]. Later, it was shown that MPN driver mutations, such as *JAK2*V617F and *MPL*, were responsible for continuous, increased signaling via the JAK2-STAT pathway and the promotion of cytokine production by malignant and non-malignant cells [58–61]. The above-mentioned hypothesis may be supported by the data concerning another *JAK2* co-expressed gene–programmed death-ligand 1 (*PD-L1*). In 2019, Guru et al. showed that *JAK2* V617F mutation was accompanied by an increased PD-L1 expression. An increased expression of *PD-L1* may be caused by excessive activation of STAT3/5 which are the regulators of PD-L1. It was also shown that in the case of *JAK2*V617F, the *PD-L1* expression was mainly mediated by STAT3 [62]. The overexpression of PD-L1 may also be caused by the acquisition of 9q UPD, which was confirmed in 6–18% of cases with ET [33, 63].

It should be noted that the PD-L1 mRNA level is not affected by the *PD-L1* or *JAK2* gene copy number variations, which has been shown in our study.

In 2020, Jacquelin et al. confirmed the mutational cooperation between the *JAK2*V617F expression and the loss of DNA methyltransferase 3A in hematopoietic cells due to monoallelic or biallelic mutations of the *DNMT3A* gene. The coexistence of the above-mentioned mutations resulted in an aberrant self-renewal, inflammatory signaling, driven by increased accessibility at enhancer elements, and finally the progression of PV to the fibrotic phase [64]. Another possibility includes the presence of other *JAK2* gene mutations affecting the mRNA splicing machinery. mRNA investigations showed no splicing defects around exon 14, and a constant level of mRNA accumulation per *JAK2* gene copy, regardless of the presence or absence of the exon 14 *JAK2*V617F mutation [65].

Another important question concerns the fluctuation of the total *JAK2* mRNA level during a natural disease outcome and evolution into the fibrotic phase (post-ET-MF). The results of our study showed a lower total *JAK2* mRNA level in post-ET-MF, in comparison to ET pts. It is in agreement with previously published data documenting lower the PD-L1 mRNA expression in *JAK2*V617F positive primary myelofibrosis in comparison to *JAK2*V617F positive ET and PV patients [33]. The detailed analysis has shown that the decrease in JAK2 and PD-L1 mRNA expression depends on the bone marrow fibrosis grade. A similar analysis concerning *JAK2*V617F VAF in ET and post-ET-MF pts did not reveal significant differences. The interpretation of the obtained data is difficult due to the fact that the fibrotic transformation in ET is rarely observed and occurs in 9–15% of pts during a long-term follow-up [66, 67]. Our data indicates that after a median time of 10 years, in patients < 60 years old, the frequency of fibrotic transformation is 8.0% (13/162 pts) which is slightly higher than reported by others (0.8–4.9%, respectively [24, 22]. An association with the applied therapy cannot be ruled out because 11.7% (19/162) of our patients were treated with anagrelide and 2.3% with PEG-interferon α [68, 69].

On the other hand, it should be noted that the clinical MPN manifestation is not only related to the type of the driver mutation but also depends on the profile of other coexisting mutations modifying the disease phenotype. The frequency and VAF of specific coexisting mutation(s) differ between pts, contributing to a specific disease phenotype in individual cases [1, 11, 70]. Among others, the mutations involved in the DNA methylation (*ASXL1*, *TET2*, *DNMT3A*, *IDH1*, *IDH2*), histone modification (*EZH2*, *ASXL1*), and splicing (*SF3B1*, *SRSF2*, *U2AF1*, and *ZRSR2*), and mutations in the transcription factors genes (*RUNX1*, *NFE2*, *PPM1D*, and *TP53*) are most frequently found in ET pts [71, 72]. An adverse prognostic relevance of some of them (*SH2B3/LNK*, *SF3B1*, *U2AF1*, *TP53*, *IDH2*, and *EZH2*) on overall, leukemia-free and myelofibrosis-free survival of ET pts was recently demonstrated [70, 73–75]. Furthermore, the *ASXL1* mutations (most frequently found in post-ET-MF pts) have been also identified as a genetic risk factor for the fibrotic transformation of ET [22]. Similarly, the co-occurrence of variants/mutations of *SRSF2* and *U2AF1* increased the risk of myelofibrotic evolution in PV and ET pts, respectively [74]. The results of our study confirmed the co-occurrence of *ASXL1*, *SRSF2*, and *U2AF1* mutations in the studied ET pts group. Their frequency was below 10% and was similar to that reported by others [73, 76]. However, it should be mentioned that its frequency in our *JAK2*V617F positive cases was 2 times higher in post-ET-MF than in ET individuals.

**Fig. 3:**
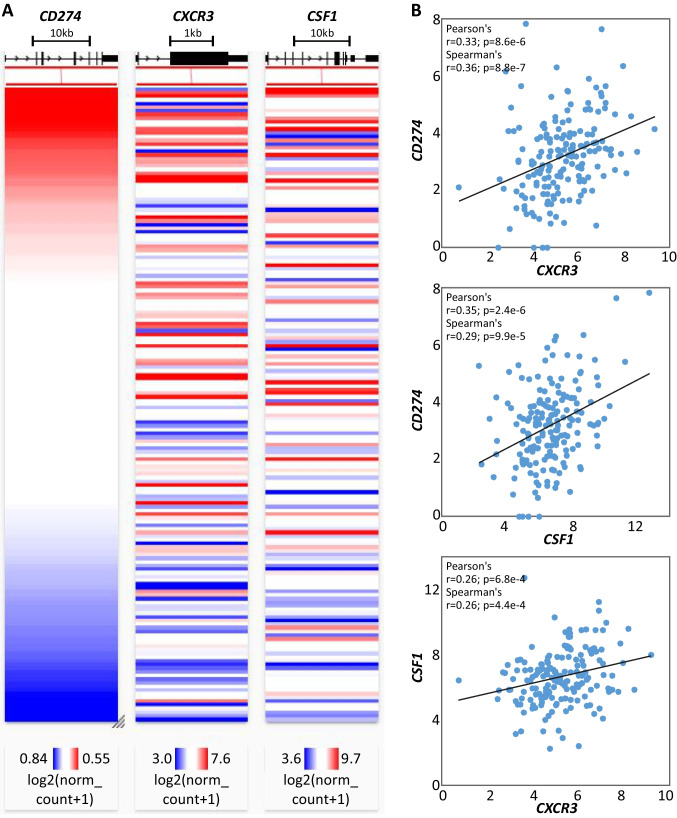
Relation between *CD274* (encoding PD-L1), *CXCR3* (encoding CXCR3), and *CSF1* (encoding M-CSF) expression levels in AML. **A** Heatmap graphs showing the expression (normalized mRNA levels; RNAseq log2(norm_count + 1)) of *CD274*, *CXCR3*, and *CSF1* genes in 173 AML samples from The Cancer Genome Atlas (TCGA) cohort (LAML). The samples were sorted according to *CD274* levels, from the highest (top, labeled in red) to the lowest (bottom, labeled in blue). The graphs visualize the overall positive relationship between levels of the three genes. **B** Scatter plots showing pairwise correlations between levels (RNAseq log2(norm_count + 1)) of the free genes; (from the top) *CC274* vs. *CXCR3*, *CD274* vs. *CSF1*, and *CXCR3* vs. *CSF1*. Each dot represents an individual AML sample. On each graph, the trend line, correlation coefficients (*r*), and *p* values for the correlations are indicated. The data were extracted, and the graphs were prepared with the use of the UCSC XENA oncogenomic portal (102)

It is, however, important to note that MPNs evolve in a biological continuum from early cancer-stages (ET, PV) to the advanced myelofibrosis stage, and other factors, including age, may also play a role [77]. Our data also indicates that the frequency of post-ET MF is age-dependent, lowest in the group of 40–60-year-old patients. Published data revealed that the C-X-C motif chemokine receptor (CXCR3) expression decreases over the MPN-continuum which may confirm the hypothesis of the reduction of the JAK-STAT induced inflammatory reaction intensity in the fibrotic disease phase [78]. The CXCR3-axis has been shown to have angiostatic and antifibrotic properties and via its chemokines’ ligands (CXCL9, CXCL10, and CXCL11) participate in the recruitment of immune/inflammatory cells: Th1 cells, CD8 + central memory T cells and effector-memory T cells, natural killer cells, natural killer T cells, plasmacytoid dendritic cells, B cells, regulatory T cells, and follicular helper T cells [79]. Recently, it was discovered that the circulating cytokine levels of B lymphocyte chemoattractant (BLC or CXCL13), TIMP metallopeptidase inhibitor 1 (TIMP-1), eotaxin-2, and macrophage colony-stimulating factor (M-CSF, previously CSF-1) reflected inflammation inside the bone marrow niche (BMN) [80, 81]. A detailed analysis of published data showed that M-CSF concentration decreases in the fibrotic phase of MPNPh- (MF > 1 vs MF ≤ 1) [81]. The mentioned changes may explain the drop in PD-L1 expression in the fibrotic phase of MPN because The Cancer Genome Atlas (TCGA) data documents a strong positive association between M-CSF, CXCR3 expression, and PD-L1 expression in another myeloid malignancy-acute myeloid leukemia (Fig. 3).

The interpretation of the simultaneous lowering of the PD-L1 and JAK2 mRNA expression detected in our study in ET patients transforming to the fibrotic phase is difficult. The bone marrow failure in the advanced ET phase is likely associated with bone marrow fibrosis, reduction of bone marrow cellularity, bone marrow myeloid metaplasia, and diversity in molecular characteristics of the emerging subclones. The latter may result in a distinct pattern of expression of *JAK2* and *PD-L1* genes [82–84]. The association between *PD-L1* expression and *JAK2*V617F mutation was recently documented by Hara et al. in a patient with a coexisting *JAK2*V617F-positive ET and lung carcinoma in whom pembrolizumab (a drug directly blocking the interaction between PD-1 and its ligands, PD-L1, and PD-L2) treatment resulted in simultaneous normalization of the platelet count and a decrease of *JAK2*V617F VAF [85].

Despite the progress in the last years in the treatment of ET, none of the available therapies can change the outcome of the disease [86]. For these reasons, there is a need to identify a new potential molecular mechanism affecting the drug resistance to improve the ET outcome. One of them is the PD1/PD-L1 axis. In 2018, Holmström et al. documented that PD-L1 specific T cell response was stronger in pts with ET and PV and weaker and rarer in pts with pre-PMF and PMF MPN [87].

The results of our study can explain, at least in part, the lack of efficacy (clinical or bone marrow pathologic response) of the pembrolizumab treatment in patients with advanced primary, post-ET-MF, and post-PV myelofibrosis [88] and shed more light on the relationship between the types of driver mutations, the PD-L1 expression, and the ET progression to the fibrotic phase.

### Study limitations

We realize that a proper distinction between ET and pre-fibrotic MF is difficult and sometimes impossible at the initial diagnosis step. We hope that the relatively long time of the follow-up and detailed monitoring of the appearance of new symptoms, characteristic for the pre-fibrotic phase of primary myelofibrosis, allowed us to omit the diagnostic pitfall in most of the ET cases. Moreover, the impact of the others factors influencing the individual ET outcome, fibrotic transformation process, and PD-L1 expression should be taken into consideration in the final interpretation of the data.

## Supplementary Information

Below is the link to the electronic supplementary material.

Supplementary file1 (DOCX 308 KB)

Supplementary file2 (DOCX 1.34 MB)

## Data Availability

All data generated or analyzed during this study are included in this published article and its supplementary information files.
