# Supplementary material for: Essential thrombocythaemia progression to the fibrotic phase is associated with a decrease in JAK2 and PDL1 levels
Source: Ann Hematol. 2022 Oct 21;101(12):2665–77. doi: 10.1007/s00277-022-05001-8 (PMC9646550; doi:10.1007/s00277-022-05001-8)
Supplement: Supplementary file 1 — Supplementary file1 (DOCX 308 KB) [file 277_2022_5001_MOESM1_ESM.docx]

**Supplementary file Methods**

Table A.

The primer sequences and PCR conditions used for the analysis of the sequence of the studied genes

| Gene/mutation | Locus  Genome Reference | Exon/intron | Primer sequence 5’-3’ | | Product size [bp] | Annealing temperature [°C] | Reference |
| --- | --- | --- | --- | --- | --- | --- | --- |
| *JAK2* V617F | LRG_612 | exon 14 | F | CTTTCTTTGAAGCAGCAAGTATGA | 101 | 60 | [33] |
|  |  |  | P | 6‐FAM‐TGAGCAAGCTTTCTCACAAGCATTTGGTTT‐TAMRA |  |  |  |
|  |  |  | R_WT | GTAGTTTTACTTACTCTCGTCTCCACAtAC |  |  |  |
|  |  |  | R_MUT | GTAGTTTTACTTACTCTCGTCTCCACAtAA |  |  |  |
| *JAK2* rs12343867 |  | intron 14 | F | ATGAAGACAAAGCATATAAATGATACA | 118 | 62 | - |
|  |  |  | R | AGTAGTTTCTGTGAACACCTAAA |  |  |  |
| *CALR* | LRG_828 | exon 9 | F | GGCAAGGCCCTGAGGTGT | 265 | 64 | [12] |
|  |  |  | R | GGCCTCAGTCCAGCCCTG |  |  |  |
| *MPL* | LRG_510 | exon 10 | F | TAGCCTGGATCTCCTTGGTG | 107 | 64 | [35] |
|  |  |  | R | GCGGTACCTGTAGTGTGCAG |  |  |  |
| *SRSF2* | LRG_640 | exon 1 | F | CCCCTCAGCCCCGTTTACC | 212 | 62 | [36] |
|  |  |  | R | TTCGCCTTCGTTCGCTTTCA |  |  |  |
| *ASXL1* | LRG_630 | exon 13 | F | AGGTCAGATCACCCAGTCAGTT | 561 | 61 | [38, 39] |
|  |  |  | R | TAGCCCATCTGTGAGTCCAACTGT |  |  |  |
| *U2AF1* | LRG_615 | exon 2 | F | GGGTGACGTCTCCCGAG | 189 | 60 | [36] |
|  |  |  | R | TCCCACCGCCTCAACCA |  |  |  |
|  |  | exon 6 | F | AATAATCAGCTCTCATTTTCCCT | 187 | 60 |  |
|  |  |  | R | ATGTAGAAATTAACTGTCTTTGAAAAGAAC |  |  |  |

***JAK2* mRNA expression assay**

The *JAK2* V617F and JAK2WT mRNA expression level determination was based on the Larssen protocol. The allele discriminating primers were modified in order to be able to bind to the cDNA sequence, since there is an intron-exon boundary adjacent to the mutation side. In order to prevent the forming of secondary structures and adjust the melting temperature of primers, two mismatches in each primer were introduced. The probe and forward primer were unmodified.

Primer R wt: GCTGAACCAGTATATTCTCGTCTCCACATAC

Primer Rmut: GCTGAACCAGTATATTCTCGTCTCCACATAA

Reverse complement **T**T**A**TGTGGAGACGAGAATAT**A**CTGGTTCAG**C MUT**

Target WT gtatgtgtctgtggagacgagaatattctggttcaggagtt

Reverse complement GT**A**TGTGGAGACGAGAATAT**A**CTGGTTCAG**C** **WT**

In order to prepare standards for the assay, additional primers were designed:

**F TTTTAAAGGCGTACGAAGAGAAGT**

**R TCAAAGGCACCAGAAAACC**

The amplification of a cDNA template with those primers generated an 834bp product. The homozygous *JAK2* V617F mutation carrier’s RNA (a patient with advanced PV) was used in order to prepare the V617F template, and a healthy donor was used for the wild type allele. The amplifications were carried for 28 cycles (linear phase), and then the products were purified (ExoSAP), diluted (1:1000) and again re-amplified for another 28 cycles. This pattern of a two-step amplification was applied in order to enrich the abundance of certain alleles and minimize the presence of another. The products were purified with ExoSap and subsequently measured with Qbit fluorimeter (Life Technologies). The measured concentration and the product length were used to calculate the copy number of the templates. The templates were diluted to 2 million copies per microliter, and then serially diluted (1:10) to obtain a standard curve. The standards were verified to check the linearity and specificity of the reaction.


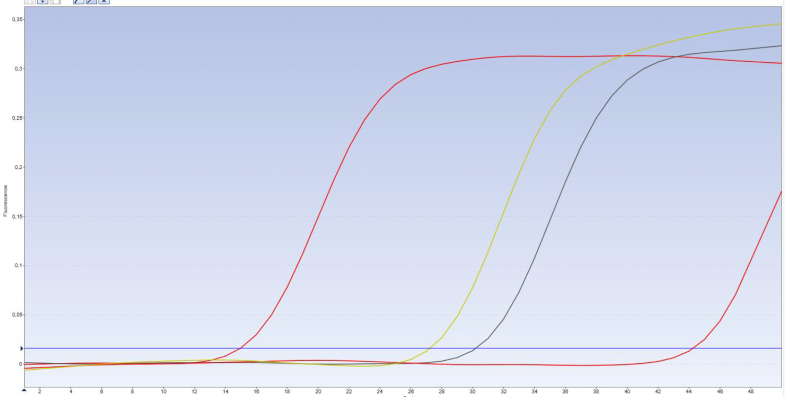


Non-specific

Specific

There was good discrimination of mutant vs. wild type allele (over 12 cycles). The specificity the other way round was significantly worse (approx. 5 cycles), but it should not significantly affect the analysis. The *GUSB* reference transcript was measured according to Gabert et al. (98).

**PD-L1 mRNA expression assay**

The PD-L1 expression was performed using Guru et al. approach(67)(65). The amplification conditions were identical to the conditions in the JAK2 expression assay (Qiagen HotStarTaq polymerase, Superscript VILO III reverse transcriptase, cDNA diluted 1:30, 50 cycles of two-step qPCR (denaturation at 95°, annealing/elongation at 60°, 50 cycles). As for the reference gene, GUSB measurements from JAK2 expression analyses were used, but instead of the copy number per reaction, the Ct values were extracted. Delta delta CT analysis of PD-L1 expression was performed according to Livak et al. (99).

**Copy number analysis of the *JAK2*, *PD-L1*, and *PD-L2* genes by the in-house designed MLPA assay**

An analysis of the copy number variation of 9p chromosome, including a densely covered region spanning the *JAK2*, *PD-L1* (*CD274*), and *PD-L2* (*PDCD1LG2*) genes, was performed with the use of an in-house designed and generated MLPA_JAK2+ assay. In total, the assay consisted of 23 probes: 6 probes specific for *JAK2*, 3 probes specific for *PD-L2*, 2 probes specific for PD-L1, 1 probe located in the region between *JAK2* and *PD-L1*, 1 probe between *PD-L1* and *PD-L2*, 5 additional probes equally distributed along the chromosome 9p arm, and 5 control probes (located on different chromosomes outside of chromosome 9 and regions of the known cancer-related genes). Among the *JAK2* specific probes, there were two V617F mutation-sensitive probes, allowing dosage quantification of the wild-type and mutant alleles. The MLPA probes and the probe-set layout were designed according to a previously proposed and well-validated strategy (44,45). Briefly, each probe was composed of two half-probes of equal size, and the total probe length ranged from 93 to 164 nt. The target sequences for the probes were selected to avoid common SNPs and repeat elements, and sequences of extremely high or low GC content. The MLPA probes were synthesized by IDT (Skokie, IL, USA). The sequences and detailed characteristics of all probes, as well as their exact positions, are schematically depicted in the Figure below and in Supplementary File 1.

The MLPA reactions were run according to the manufacturer’s general recommendations (MRC-Holland, Amsterdam, the Netherlands) and described in the seminal study (100). All reagents except the probe mixes were purchased from MRC-Holland (http://www.mlpa.com). The products of the MLPA reaction were subsequently diluted 20x in HiDi formamide containing GS Liz600, which was used as a DNA sizing standard and separated according to their size with capillary electrophoresis (POP7 polymer) in an ABI Prism 3130XL apparatus (Applied Biosystems, Carlsbad, CA, USA). The obtained electropherograms were analyzed using GeneMarker software v2.4.0 (SoftGenetics, State College, PA, USA). The probe signals (peak heights) were used for copy number and the V617F alleles quantifications, as described earlier (45). Briefly, for each individual sample, the signal intensity of each probe was divided by the average signal intensity of the control probes to normalize run-to-run (sample-to-sample) variation. The normalized signal of each probe in the tested samples was divided by the corresponding signal in the reference sample (a sample with no copy number change in the analyzed positions) and multiplied by 2. The obtained values that corresponded with the copy number of particular regions were visualized in bar graphs and the dosage of V617F mutation was expressed as a percentage of the mutant allele.


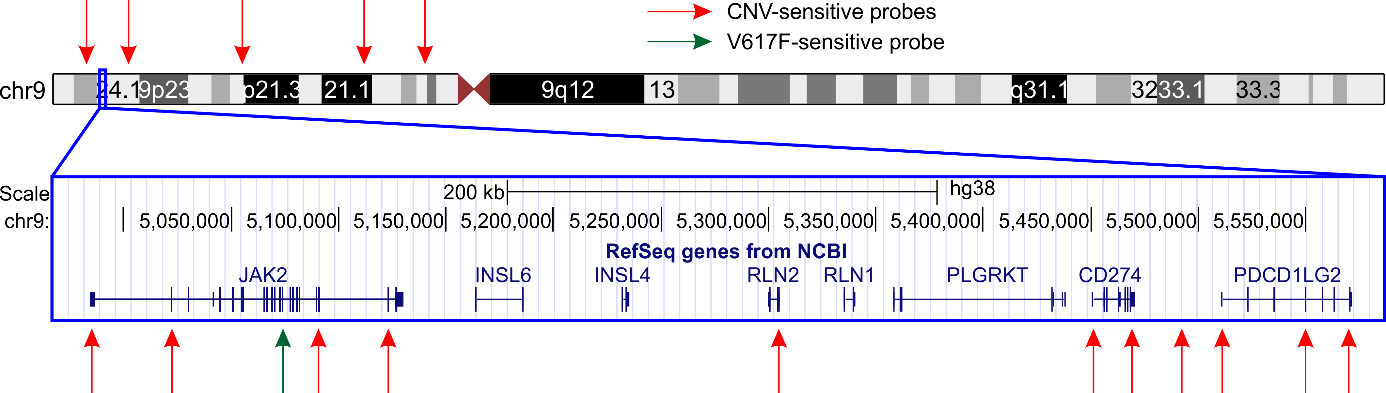


Figure. Scheme of the copy number analysis of the *JAK2*, *PD-L1*, and *PD-L2* genes by the in-house designed MLPA assay. The copy number variation probes are indicated by red arrows, the JAK2 V617F specific probe by a green arrow
