# Supplementary material for: Essential thrombocythaemia progression to the fibrotic phase is associated with a decrease in JAK2 and PDL1 levels
Source: Ann Hematol. 2022 Oct 21;101(12):2665–77. doi: 10.1007/s00277-022-05001-8 (PMC9646550; doi:10.1007/s00277-022-05001-8)
Supplement: Supplementary file 2 — Supplementary file2 (DOCX 1.34 MB) [file 277_2022_5001_MOESM2_ESM.docx]

**Supplementary file Results**

Figure A. Hemoglobin concentration in the blood of the studied patients according to different driving mutation subtypes of ET (n=162)


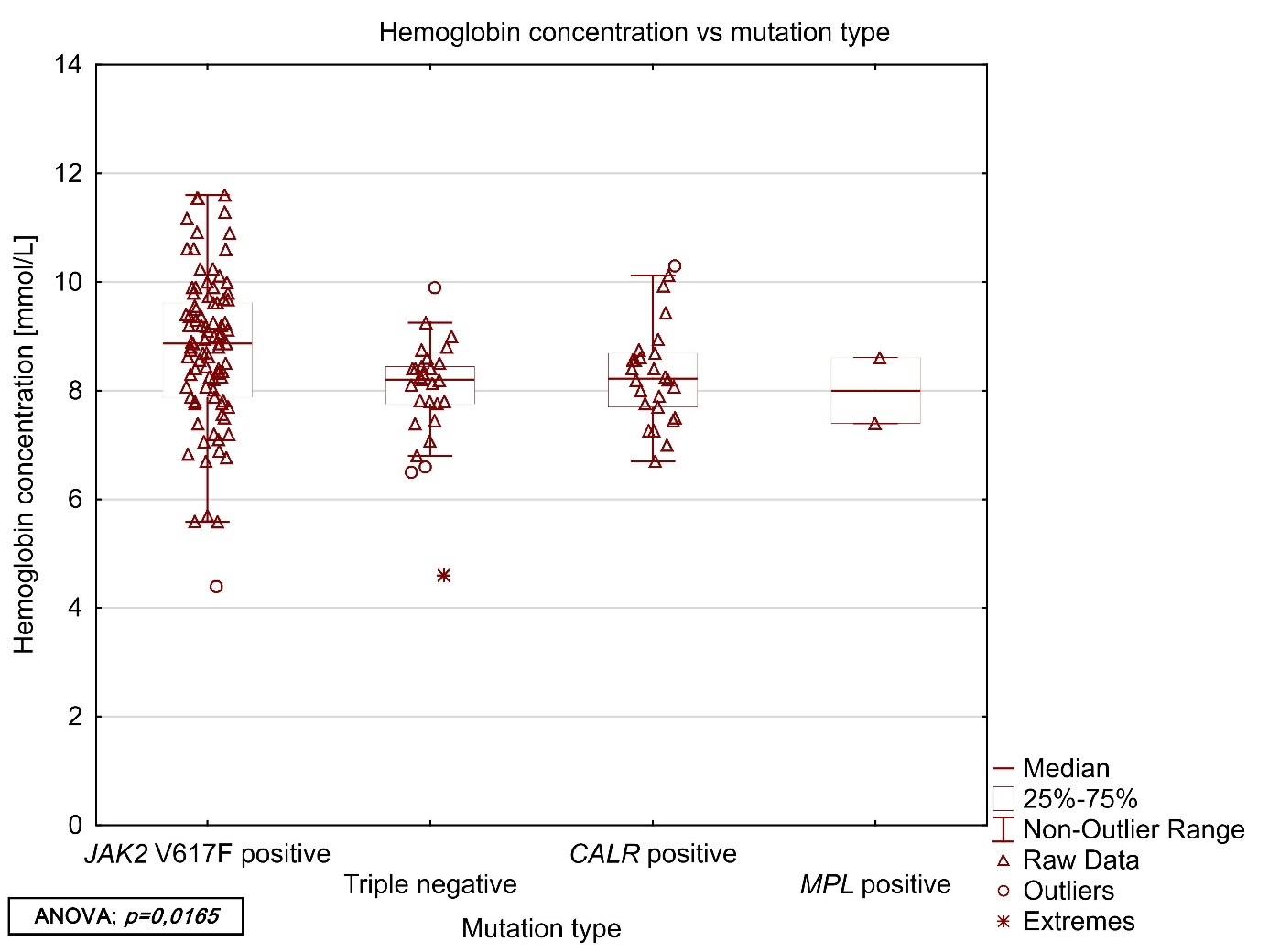


Figure B. The comparison of hemoglobin concentration in ET (n=132) and post-ET-MF (n=30) patients


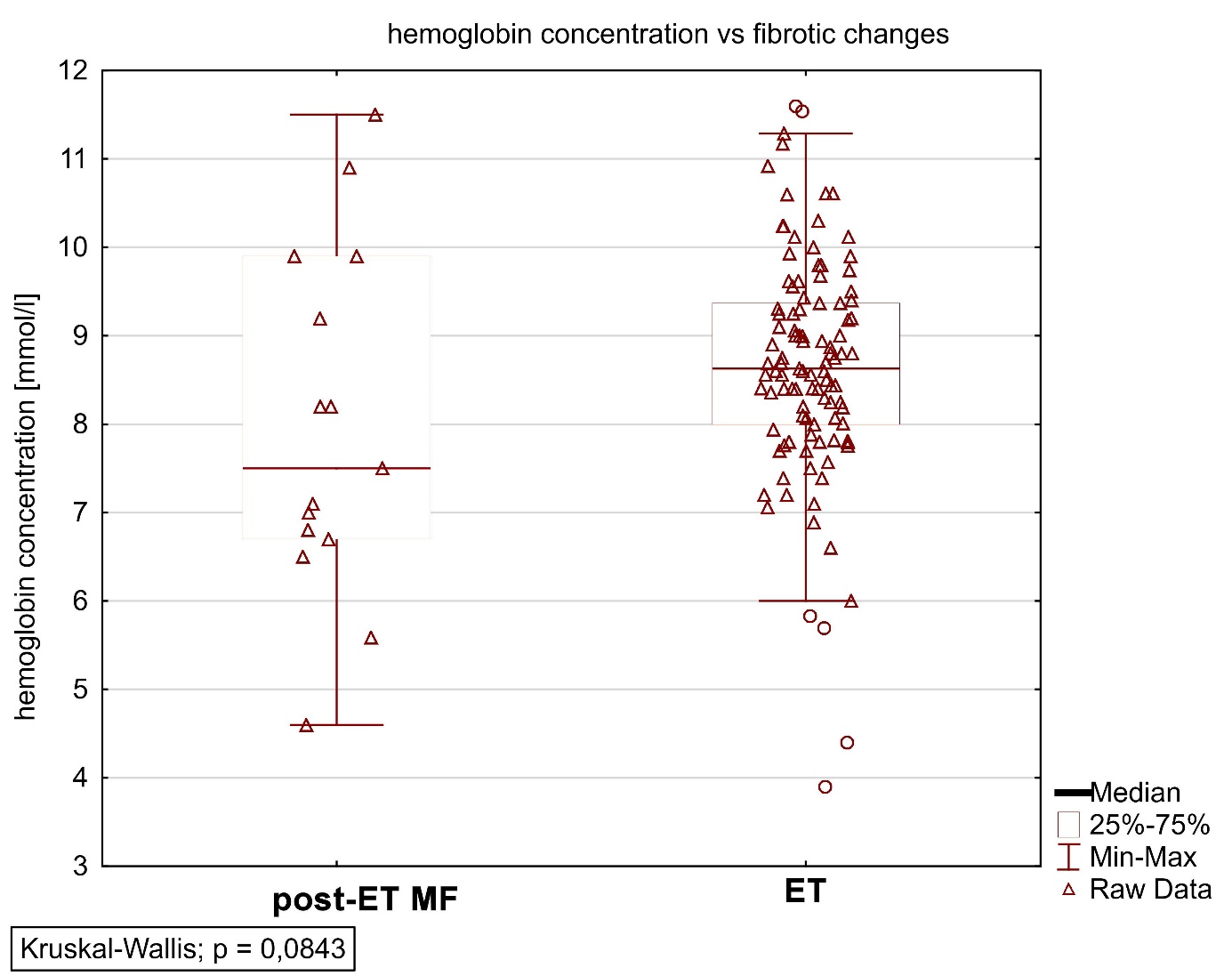


Figure C. The leukocyte count (WBC) in ET patients (n=162) depending on the *JAK2*V617F allele burden [low (≤50%) vs high (>50%)]


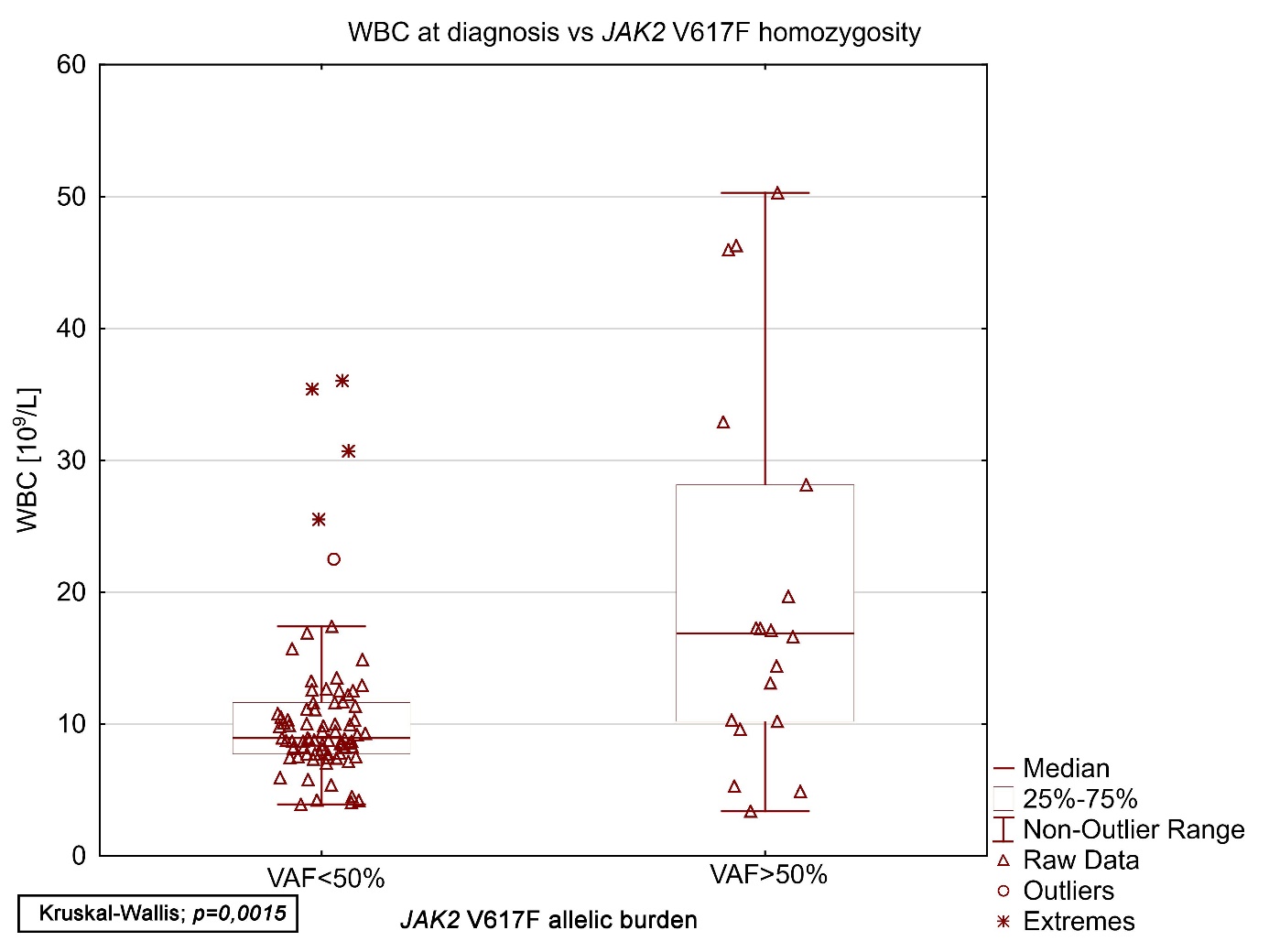


Figure D. The median platelet count in ET patients depending on the *CALR* mutation status


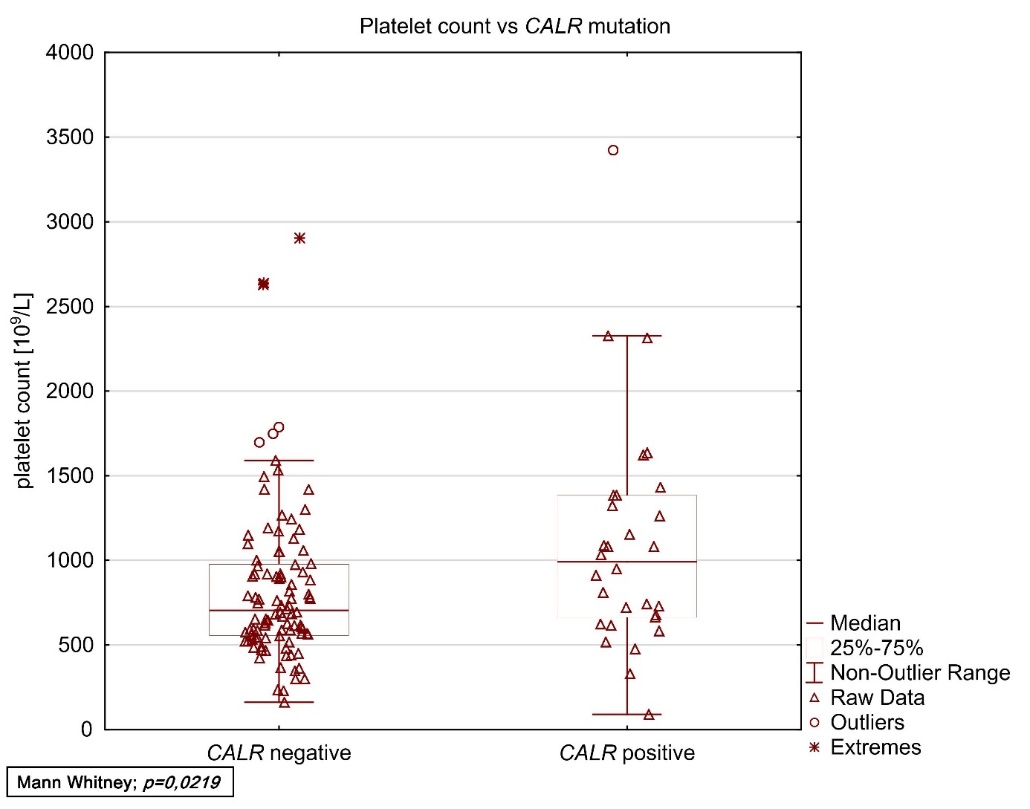


Figure E. The *JAK2*V617F VAF distribution in different the JAK2 haplotype^GGCC_46/1^ groups of ET patients (n=162)


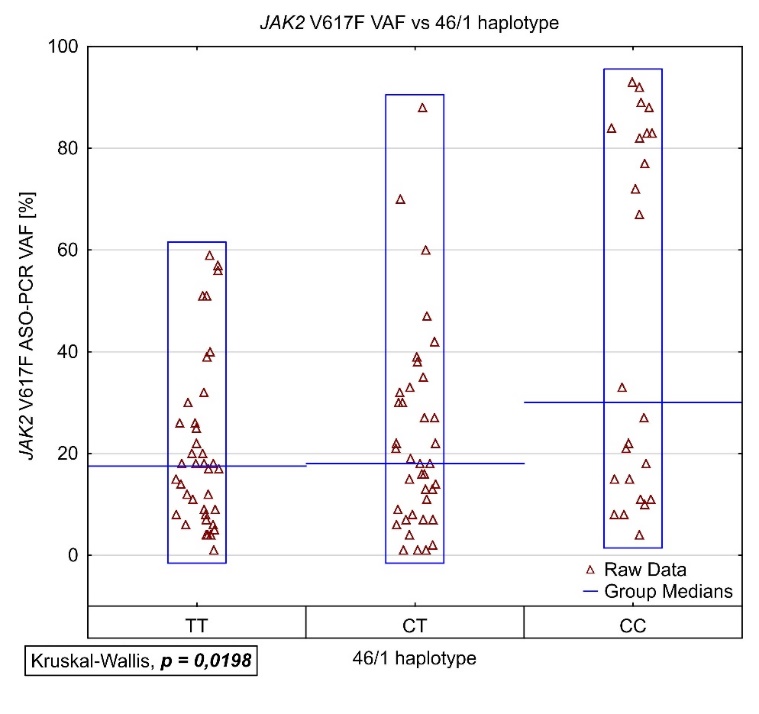
B.


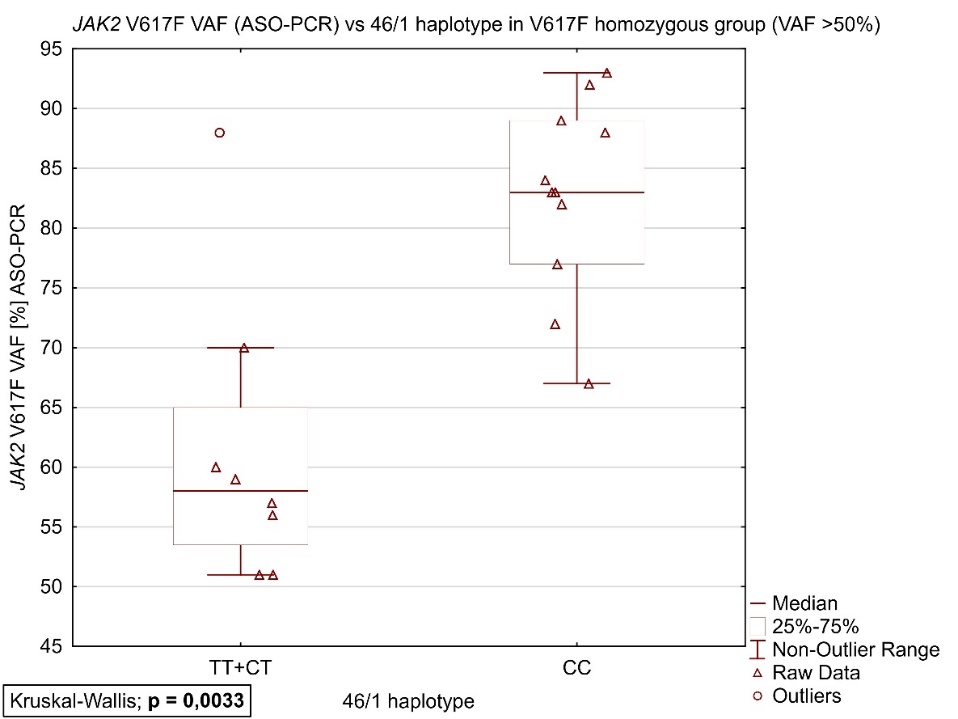


Figure F. The correlation between the *JAK2*V617F expression and the *JAK2*V617F VAF in ET patients


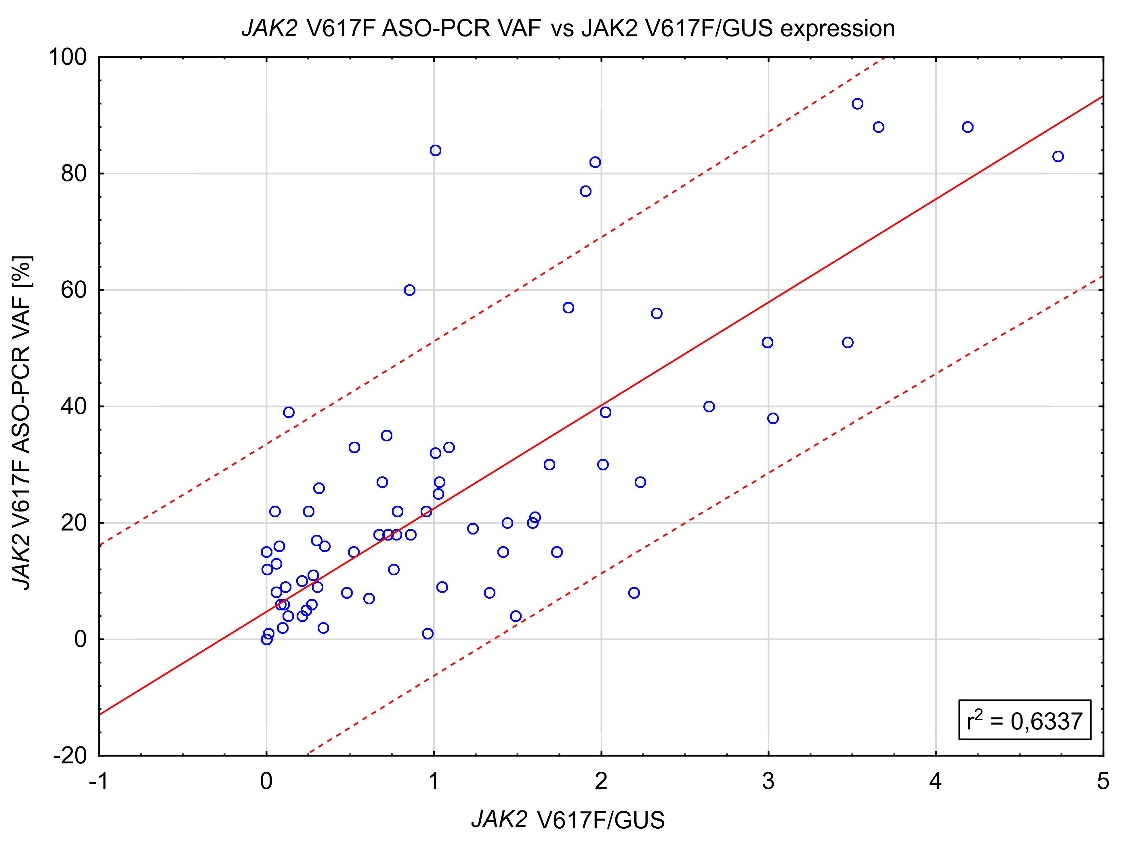


Figure G. The total level of *JAK2* mRNA ET patients with and without *JAK2*V617F mutation


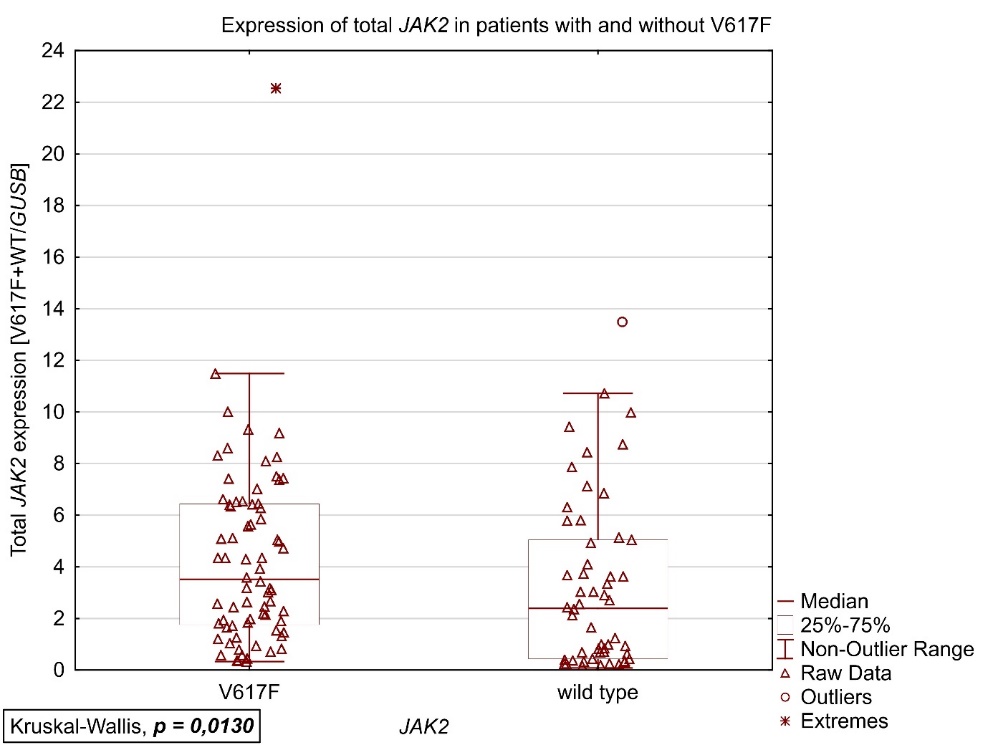


Figure H. The correlation between the *JAK2*V617F VAF determined by ASO-PCR method and MLPA in ET patients (n=162)


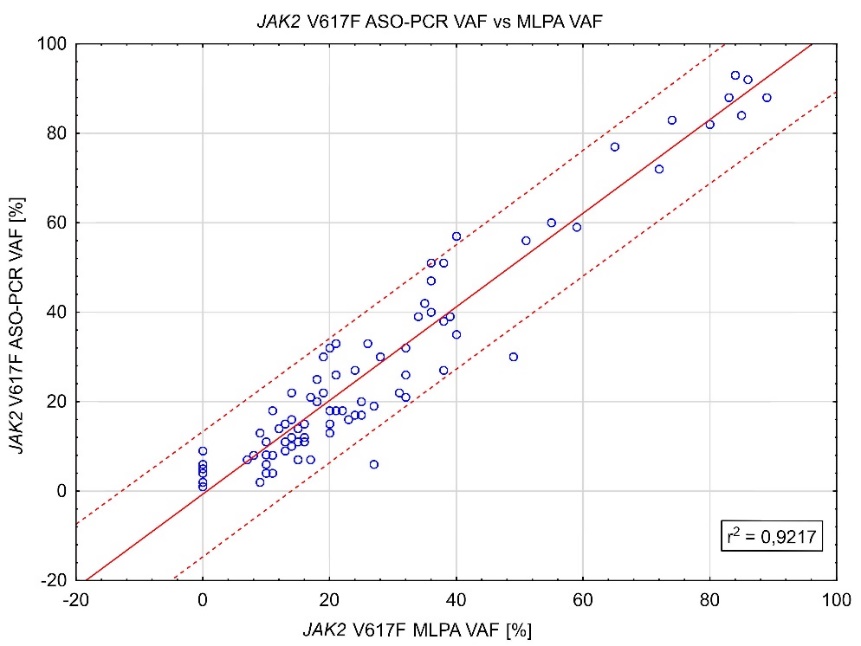


Figure I. Total *JAK2* mRNA level in the ET patients with the different types of driver mutation


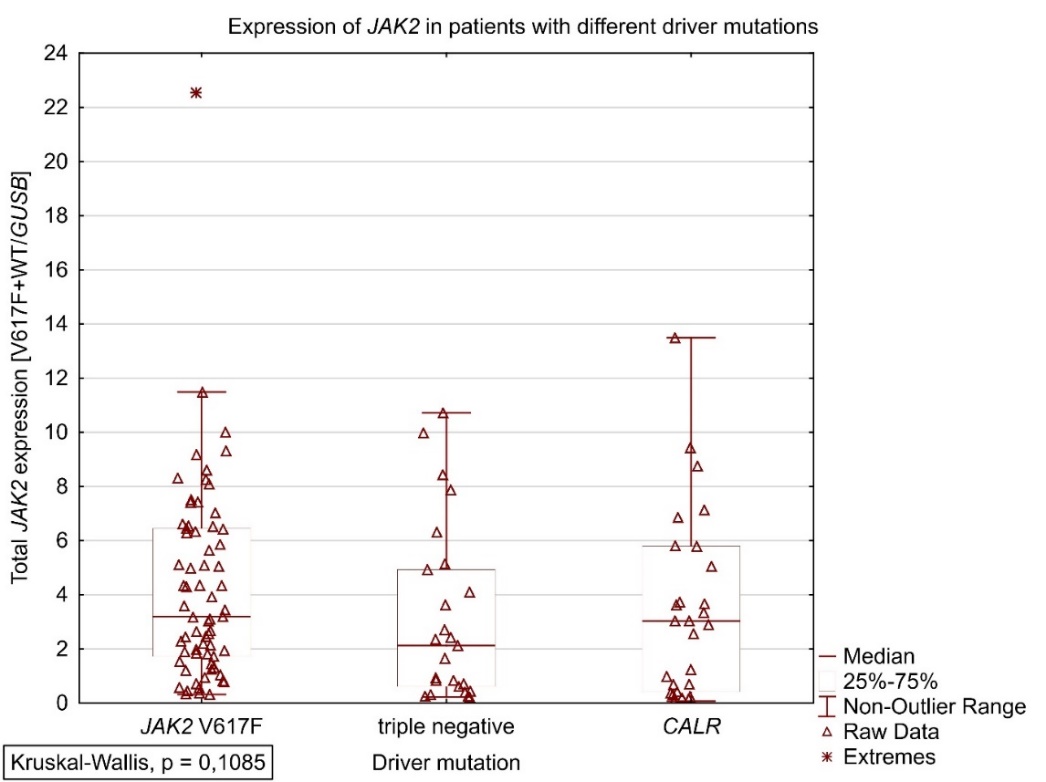


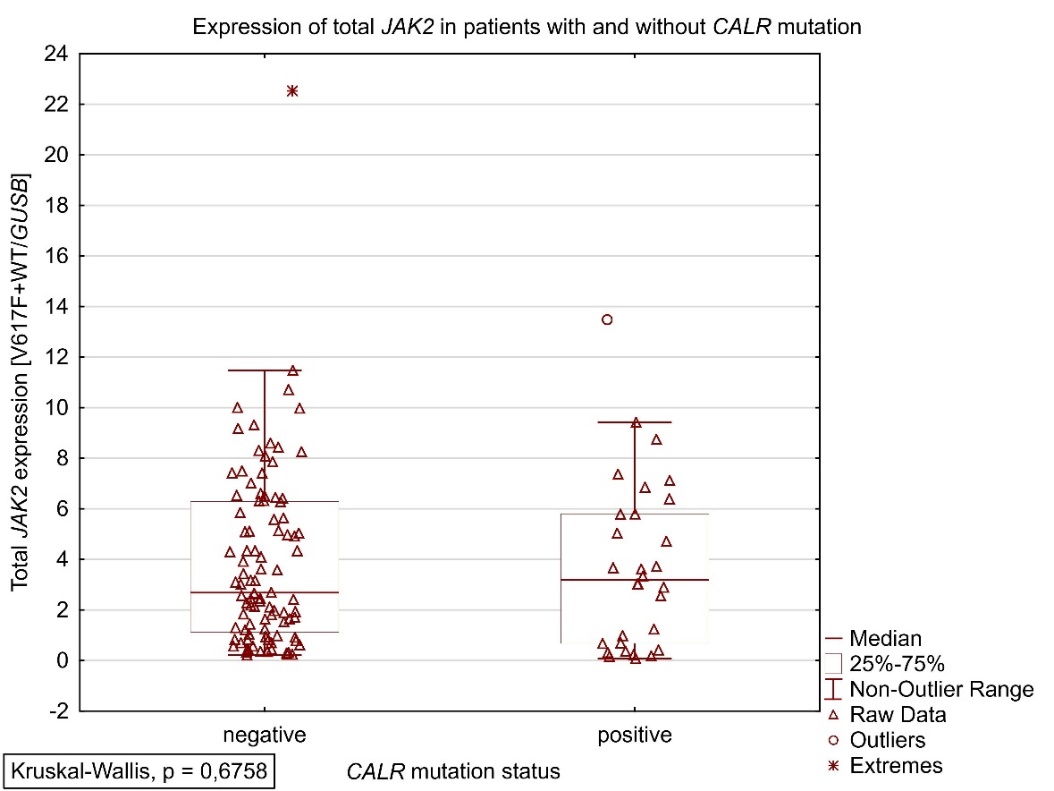


Supplementary Table A. Driver and non-driver gene mutations in ET (n=164) and post-ET-MF (n=32)

| Gene | Nucleotide change | Protein change | n | Remarks |
| --- | --- | --- | --- | --- |
| *JAK2* | c.1849G>T | p.Val617Phe | 100 |  |
| *CALR* | c.1099_1150del | p.Leu367Thrfs*46 | 13 |  |
|  | c.1154_1155insTTGTC | p.Lys385Asnfs*47 | 17 |  |
|  | c.1154_1155insATGTC | p.Glu386Cysfs*46 | 1 |  |
|  | c.1099_1144del | p.Leu367Argfs*48 | 1 |  |
|  | c.1124_1142del | p.Lys375Argfs*49 | 1 |  |
|  | c.1101_1134del | p.Lys368Argfs*51 | 1 |  |
| *MPL* | c.1544G>T | p.Trp515Leu | 3 | In two patients, the identification of the type mutation was impossible due to a low mutation allele burden (positive HRMA signal) |
| *SRSF2* | c.284C>A | p.Pro95His | 1 |  |
|  | c.284C>T | p.Pro95Leu | 1 |  |
|  | c.284C>G | p.Pro95Arg | 1 |  |
| *ASXL1* | c.1934dup | p.Gly646Trpfs*12 | 4 |  |
|  | c.1936G>A | p.Gly646Ser | 1 |  |
|  | c.2110G>T | p.Gly704Thr | 1 |  |
| *U2AF1* | c.101C>T | p.Ser34Phe | 2 |  |
|  | c.470A>G | p.Gln157Arg | 2 |  |

Abbreviations: ET- essential thrombocythaemia, post-ET-MF- post essential thrombocythaemia myelofibrosis, *CALR*- calreticulin gene, *MPL*- thrombopoietin receptor gene, *ASXL1*- additional sex comb-like gene, SRSF2- serine and arginine rich splicing factor 2 gene, *U2AF1* – U2 small nuclear RNA auxillary factor 1 gene

Table B. Distribution of non-driver gene mutations in patients with ET and post-ET-MF according to the initial mutational status

|  | **Driver mutation** | ***ASXL1*** | ***SRSF2*** | ***U2AF1* Q157** | ***U2AF1 S34*** |
| --- | --- | --- | --- | --- | --- |
| ET  (n=132) | *JAK*2V617F | 3 | 2 | - | 2 |
|  | *CALR* type1 | - | - | - | - |
|  | *CALR*-type 2 | - | 1 | - | - |
|  | *CALR* others | - | - | - | - |
| Post-ET-MF  (n=30) | *JAK*2V617F | 2 | - | 1 | - |
|  | *CALR* type1 | 1 | - | 1 | - |
|  | *CALR*-type 2 | - | - | - | - |
|  | *CALR* others | - | - | - | - |

Abbreviations = ET–essential thrombocythaemia, post-ET-MF–post-essential thrombocythaemia myelofibrosis, *CALR* – calreticulin gene, *MPL*–thrombopoietin receptor gene, *ASXL1* – Additional sex comb-like 1 gene, *SRSF2 –* Serine and arginine rich splicing factor 2 gene, *U2AF1* –U2 small nuclear RNA auxiliary factor 1 gene

| **Diagnosis** | **Patient’s ID** | ***JAK2* V617F** | ***JAK2*V617F allele burden (%)** | ***CALR*** | ***MPL*** | ***ASXL1*** | ***SRSF2*** | ***U2AF1*** |
| --- | --- | --- | --- | --- | --- | --- | --- | --- |
| Post-ET-MF | 296/14 | **+** | 35 | - | **-** | c.1934dup p.Gly646Trpfs*12 | **-** | **-** |
|  | 354/16 | **+** | 1 | - | **-** | c.1934dup p.Gly646Trpfs*12 | **-** | c.470A>G p.Gln157Arg |
|  | 271/17 | **-** |  | c.1099_1150del p.Leu367Thrfs*46 | **-** | c.1934dup p.Gly646Trpfs*12 | **-** | **-** |
|  | 367/15 | **-** |  | c.1099_1150del p.Leu367Thrfs*46 | **-** | - | **-** | c.470A>G p.Gln157Arg |
| ET | 117/11 | **+** | 32 | - | **-** | - | **-** | c.101C>T p.Ser34Phe |
|  | 96/15 | **+** | 57 | - | **-** | - | c.284C>A p.Pro95His | **-** |
|  | 236/18 | **-** |  | c.1154_1155insTTGTC p.Lys385Asnfs*47 | **-** | - | c.284C>T p.Pro95Leu | **-** |
|  | 491/18 | **+** | 16 | - | **-** | - | c.284C>G p.Pro95Arg | **-** |
|  | 163/16 | **+** | 19 | - | **-** | c.1936G>A  p.Gly646Ser | **-** | c.101C>T p.Ser34Phe |
|  | 853/17 | **+** | 38 | - | **-** | c.1934dup p.Gly646Trpfs*12 | **-** | **-** |
|  | 16/18 | **+** | 2 | c.1099_1150del p.Leu367Thrfs*46 | **-** | c.2110G>T  p.Gly704Thr | **-** | **-** |
|  | 156/19 | **+** | 1 | c.1154_1155insTTGTC p.Lys385Asnfs*47 | **-** | **-** | **-** | **-** |
|  | 317/19 | **+** | 12 | c.1154_1155insTTGTC p.Lys385Asnfs*47 | **-** | **-** | **-** | **-** |
|  | 429/16 | **+** | 1 | - | **+^a^** | **-** | **-** | **-** |

Table C. Characteristics of ET (n=132) and post-ET-MF (n=30) patients with two or more mutations in the tested genes

^a^- detected using HRM only

Abbreviations = ET– essential thrombocythaemia, post-ET-MF– post- essential thrombocythaemia myelofibrosis, *CALR* – calreticulin gene, *MPL* – thrombopoietin receptor gene mutation, *ASXL1* – Additional sex comb-like 1 gene, *SRSF2 –* Serine and arginine-rich splicing factor 2 gene, *U2AF1* – U2 small nuclear RNA auxiliary factor 1 gene

Table D. Z-score analysis of the occurrence of different 46/1 haplotype in ET patients (n=162) dependently from driver mutation status

|  | Driver mutation | | |
| --- | --- | --- | --- |
| Haplotypes | *JAK2* V617F vs WT | *CALR* | Triple-negative |
| C/C vs C/T+T/T | **.0228** | 0,1094 | 0,1087 |
| C/T vs C/C+T/T | 0,35 | 0,3803 | 0,3108 |
| T/T vs C/C+C/T | 0,098 | 0,248 | 0,2973 |
